# Beam Output Audit results within the EORTC Radiation Oncology Group network

**DOI:** 10.1186/s13014-016-0733-4

**Published:** 2016-12-15

**Authors:** Coen W. Hurkmans, Melissa Christiaens, Sandra Collette, Damien Charles Weber

**Affiliations:** 1 Department of Radiation Oncology, Catharina Hospital, Eindhoven, The Netherlands; 2 EORTC HQ, Brussels, Belgium; 3 Clinic for Particle Therapy, West German Proton Therapy Centre Essen, University Hospital Essen, Essen, Germany; 4 EORTC ROG RTQA Strategic Committee, EORTC, Brussels, Belgium; 5 Centre for Proton Therapy, Paul Scherrer Institute, Villigen, Switzerland

**Keywords:** Quality assurance, Beam output, Radiotherapy, Clinical trial, Audit

## Abstract

Beam Output Auditing (BOA) is one key process of the EORTC radiation therapy quality assurance program. Here the results obtained between 2005 and 2014 are presented and compared to previous results.

For all BOA reports the following parameters were scored: centre, country, date of audit, beam energies and treatment machines audited, auditing organisation, percentage of agreement between stated and measured dose.

Four-hundred and sixty-one BOA reports were analyzed containing the results of 1790 photon and 1366 electron beams, delivered by 755 different treatment machines. The majority of beams (91.1%) were within the optimal limit of ≤ 3%. Only 13 beams (0.4%; *n* = 9 electrons; *n* = 4 photons), were out of the range of acceptance of ≤ 5%. Previous reviews reported a much higher percentage of 2.5% or more of the BOAs with >5% deviation.

The majority of EORTC centres present beam output variations within the 3% tolerance cutoff value and only 0.4% of audited beams presented with variations of more than 5%. This is an important improvement compared to previous BOA results.

## Introduction

In 1982 the European Organisation for Research and Treatment of Cancer - Radiation Oncology Group (EORTC-ROG started a quality assurance (QA) program in radiation therapy (RTQA). ) In 1986 a Beam Output Audit (BOA) program was incorporated in this RTQA program [1, 2]. This RTQA process is in essence a verification of the dose delivery under reference conditions at the accruing site and defines, with the facility questionnaire the RTQA Level 1 [3]. It is a dose measurement performed by a national or international auditor, independent from the site, and the results are fed back to EORTC headquarters [4]. Published BOA data measured by an independent body and collected in the perspective of a trial coordination organization is scarce. A description of the method with mailed Thermoluminescence dosemeters (TLDs) and the results for the 1987–1989 and 1987–1992 periods have been published earlier [1, 5]. Themain goal of this paper is to present the BOA results from 2005 to 2014 stemming from European and non-European centres participating in EORTC trials.

## Materials and methods

There are a number of EORTC-ROG minimum requirements that centres should fulfill to get their BOA approved by the EORTC-ROG [3]. First and foremost, the audit must be performed independently from the centre, including at least the highest and lowest photon energy. The date of measurement should not be longer ago than 2 years at the time a request to participate in a new trial is received from a centre by the EORTC. Flattened beams and flattening filter free beams are considered as different energies. Electron beams are optional, unless the centre wants to participate in a study with electron treatments. All measurements need to be preferably within the 3% agreement, but measurements up to and including 5% are also accepted [3]. The institutions are notified of the decision of the EORTC and are alerted if beams are within the 3 to 5% range if applicable. If a site does not fulfil the 5% tolerance for a single machine or beam the site is not authorized to use that combination of treatment unit type and energy. The EORTC currently distinguishes 3 treatment unit types: standard linear accelerator, rotational and robotic. The auditor’s primary standard has to have a clear traceability back to a national or international primary standard and the measurements must take place under standard conditions.

BOA reports capture the following parameters: centre, country, date of audit, beam energies and treatment machines audited, auditing organisation and the measured and stated dose. In addition to the mandatory measurements under standard conditions, a commercial service provider (called EQUAL) also reported the measurements they performed on the beam axis under non-standard conditions (wedged beams, field size 15x20cm, field size 7x7cm, at SSD of 105, 110 and 115 cm). Additionally, the number of treatment machines was collected for each centre based upon the latest EORTC facility questionnaire.

## Results

**Fig. 1:**
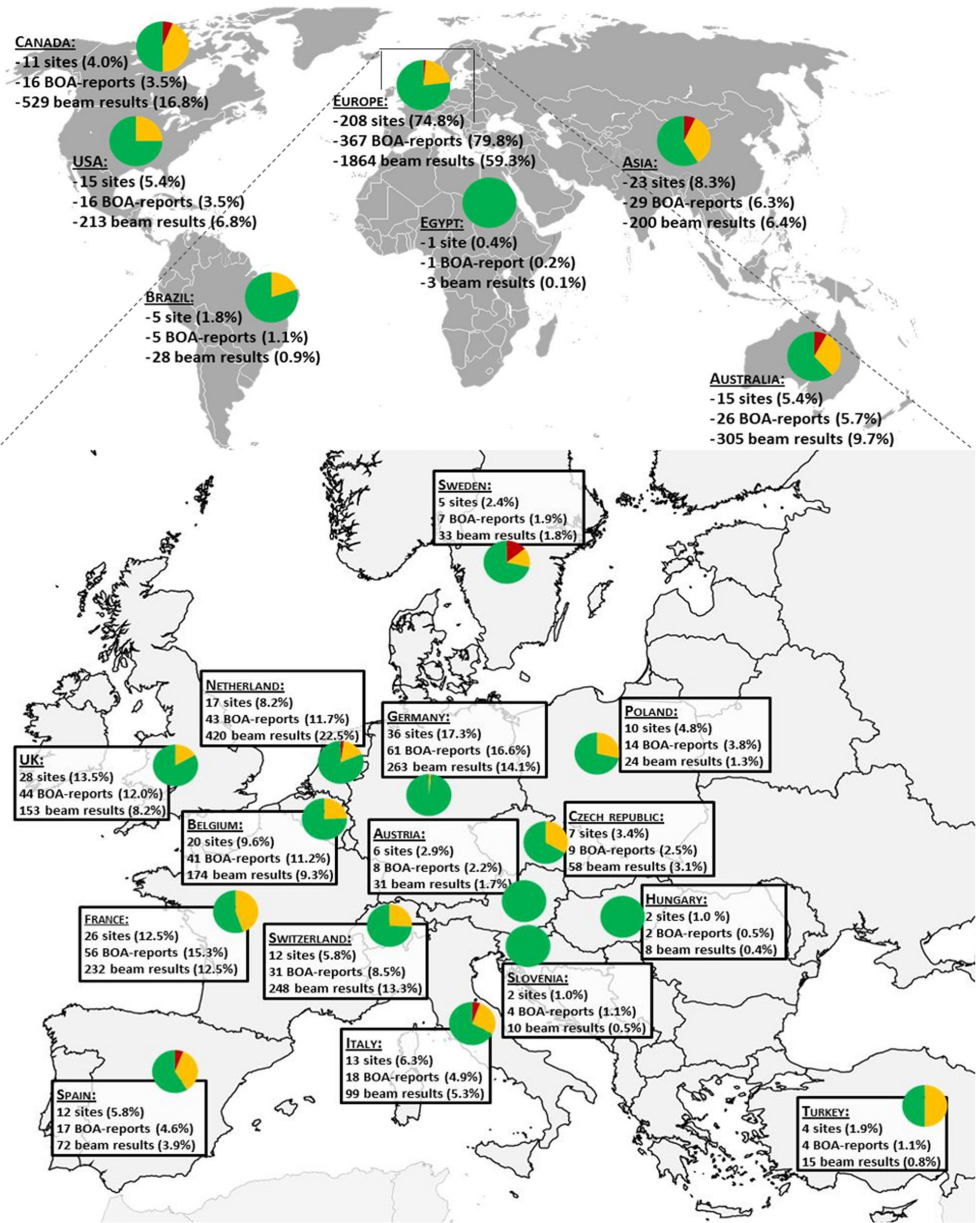
World-wide distribution and focus on European distribution of centres providing BOA-reports collected and stored at the EORTC. Not taken into account are the Co60 beams and the ‘stereotactic radiosurgery dosimetry’. The ‘pie-diagrams’ represent the subdivision between the different levels of acceptance [*green*: ≤3%; *yellow*: >3% and ≤ 5%; *red*: >5%]

A total of 461 BOA-reports, performed over a time period of 107 months (February 2005 until December 2013) were assessed. These reports were made for 279 centres worldwide, stemming from 33 countries, which provided at least one BOA-report to EORTC. The results of 3174 beams, delivered by 755 different treatment units were assessed. This included 18 results (0.6%) for Co-60 beams, 1790 (56.4%) for photon beams (of which 11 ‘stereotactic radiosurgery dosimetry’) and 1366 (43.0%) for electron beams. The world-wide distribution of the centres providing BOA-reports and the subdivision for each region by acceptance level is shown in Fig. 1. Not presented in the figure of Europe are Luxembourg, Slovakia, Lithuania, Norway, Denmark, Portugal, Cyprus and Ireland, which each have one BOA-report and were all within the optimal level of acceptance, except Ireland and Lithuania, which had a report within the limit of 5% and Portugal, which had a report out of the limit of 5%. The majority (64%) of centres submitted 1 BOA report. Forty-four centres (16%) submitted a BOA-report twice and 55 (20%) more than twice over the 9-year study period.

Most of the audits were performed by EQUAL. Most other different auditing bodies can be brought back to the common denominator of national organizations.

For all but 4 (1.4%) centres, the number of treatment units at the radiation therapy centre could be retrieved. The number of treatment units present at a centre ranged from 1 to 17, with a median of 4. Ten institutions provided reports which did not fulfill the EORTC requirements. Therefore, the following results will include the valid 451 BOA reports, excluding the 18 Co-60 beams, of which 17 were within the optimal 3% limit and 1 between 3 and 5%. The results are summarized in Table 1.

**Table 1:** Summary of all beam results

Cutoff level	Photon beams (%)	Electron beams (%)	Co-60 beams
<3%	1670 (94.4%)	1225 (89.9%)	17
3–5%	96 (5.4%)	129 (9.5%)	1
>5%	4 (0.2%)	9 (0.7%)	
Total (451 BOA reports)	1770 (100%)	1363 (100%)	18

**Fig. 2:**
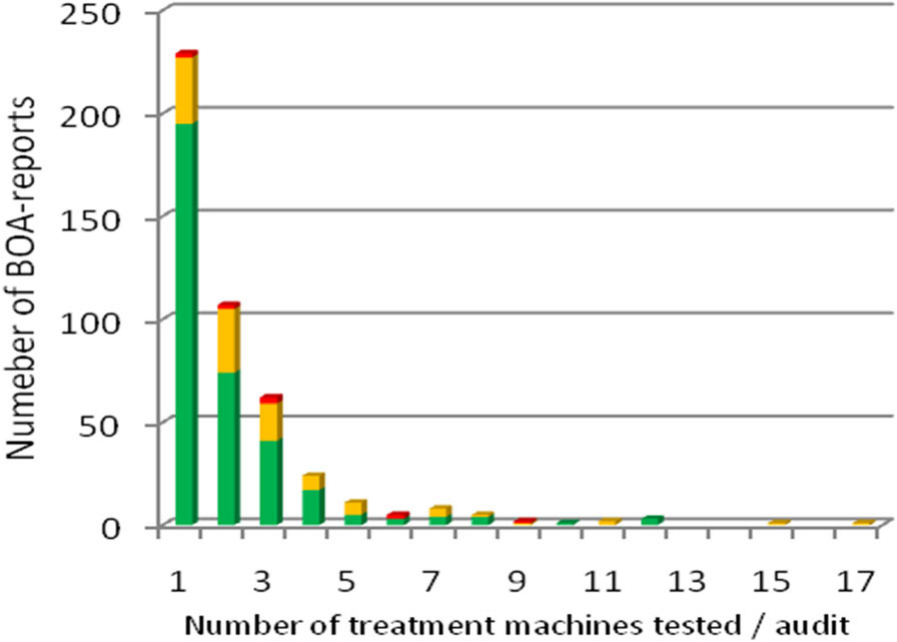
Number of beams per audit, subdivided between the different levels of acceptance [*green*: ≤3%; *yellow*: >3% and ≤ 5%; *red*: >5%]

**Fig. 3:**
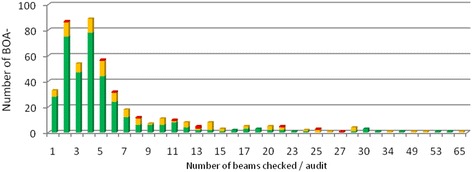
Number of treatment units per audit, subdivided between the different levels of acceptance [*green*: ≤3%; *yellow*: >3% and ≤ 5%; *red*: >5%]

**Fig. 4:**
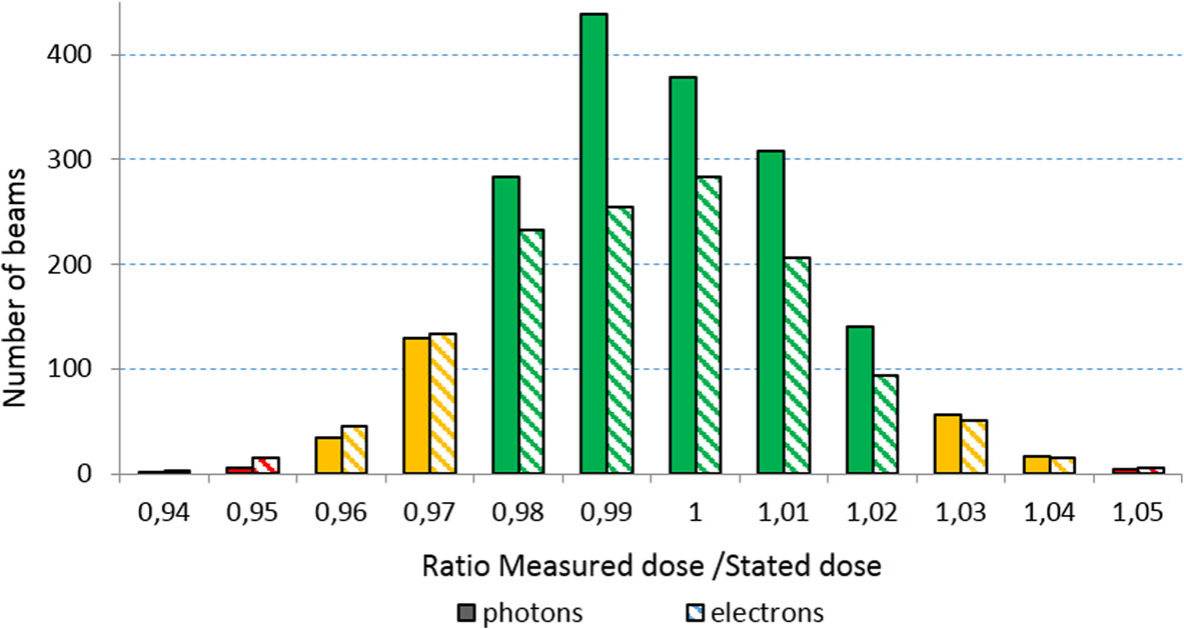
Presentation of all beam-results, with a subdivision in photon- and electron beams. The colors differentiate between the levels of acceptance [*green*: ≤3%; *yellow*: >3% and ≤ 5%; *red*: >5%]

During an audit, the median number of beams tested was 4 (range: 1–65) (Fig. 3), the median number of treatment units was 2 (range: 1–17) (Fig. 2) and the median number of beams tested per treatment unit was 2.8 (range: 1 – 9). The median of the ratio of the number of machines tested and number of machines in the centre was 0.4 (range: 0.08-1). Thirteen beams (0.4%; *n* = 9, electrons; *n* = 4, photons) were out of the range of acceptance of 5%, 225 beams (8.5%; 129 electrons and 96 photons) were within the non-optimal range of acceptance (3-5%) and 2895 (91.1%; 1225 electrons and 1670 photons) were within the optimal limit of ≤ 3%. An absolute average difference between stated and measured dose of 1.35% for photons (SD: 0.99) and 1.55% for electrons (SD: 1.16) could be seen. The average ratio of the measured dose and stated dose (Dm/Ds) was 1.00 for photons and 0.99 for electrons (Figs. 3 and 4). The 13 unacceptable beams were from ten BOA-reports, coming from 8 centres.

**Fig. 5:**
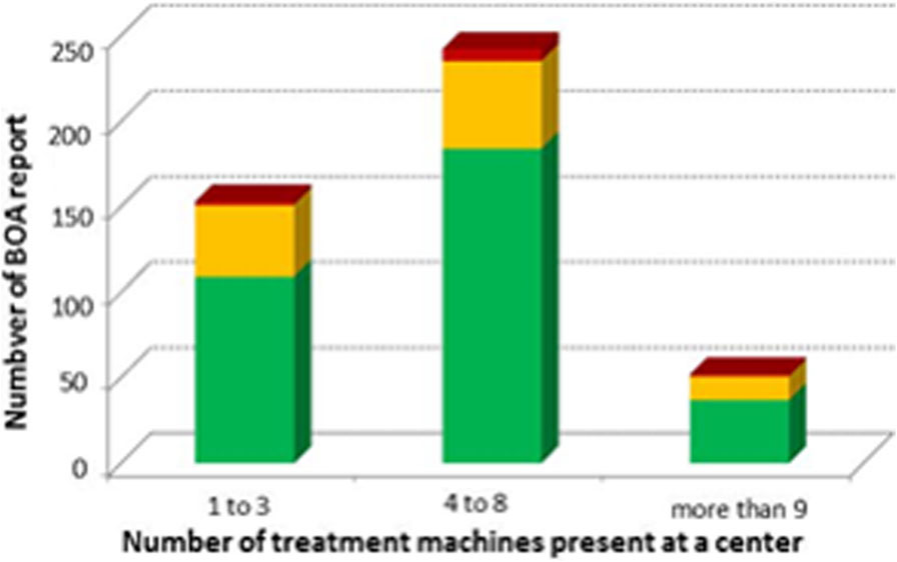
Number of BOA-reports subdivided in the level of acceptance per number of treatment machines present at a centre. The colors differentiate between the levels of acceptance [*green*: ≤3%; *yellow*: >3% and ≤ 5%; *red*: >5%]

**Fig. 6:**
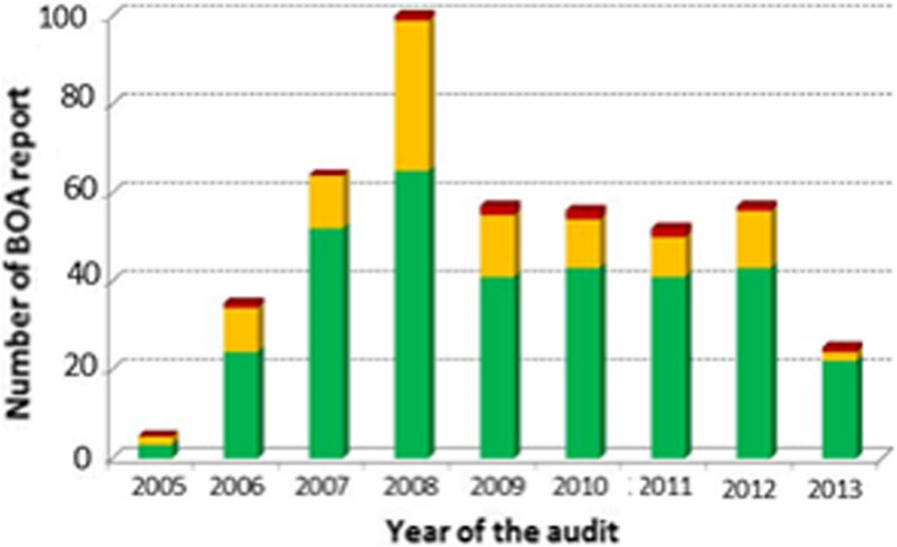
Number of BOA-reports, subdivided in level of acceptance per year of audit. The colors differentiate between the levels of acceptance [*green*: ≤3%; *yellow*: >3% and ≤ 5%; *red*: >5%]

Out of 269 centres, 180 (66.9%) had all beams within the optimal acceptance level (≤3%) and 81 (30.1%) had at least one beam result within the non-optimal level, but none out of the range of acceptance. One might expect that larger centres, having more experience in beam commissioning and beam QA, would have better BOA results than smaller centres. However, no association was observed between the BOA results and the number of treatment units at a centre. (Fig. 5). In addition, no association could be seen with the audit year (Fig. 6). For the centres, which provided more than one BOA-report, the evolution over time was checked and no clear improvement of the results over the reported time period for the different centres was seen.

The reports of the audits conducted by EQUAL also contained measurements in non-standard conditions. At the 3% cutoff level, the pass-rates were for photons 92.2% (standard) vs 93.7% (non-standard) (*p* = 0.35, chi-square test of 2 proportions) and for electrons 87.4% (standard) vs 84.8% (non-standard) (*p* = 0.39). No comparison for these pass-rates were made at the 5% cutoff level as they contained too few results. Overall, 6.5% of the photon beams measured by EQUAL were between 3–5 and 0.1% above 5%. More details about the photon beam results from the EQUAL measurements can be found in Table 2.

**Table 2:** Photon beam results from the EQUAL audits for non-standard conditions

Cutoff level	Percentage depth dose (%)	Beam output variation open beams (%)	Beam output variation wedged beams (%)	Wedge transmission factor
<3%	242 (93%)	462 (93%)	84 (88%)	215 (95%)
3-5%	15 (7%)	29 (5%)	11 (12%)	11 (5%)
>5%	0 (0%)	2 (1%)	0 (0%)	0 (0%)
Total	257 (100%)	493 (100%)	95 (100%)	226 (100%)

## Discussion

When comparing the current results with earlier reported results an important difference can be seen. An evaluation over a time period of 6 years (1987–1992), auditing 55 institutes showed 2.5% of the beams with a higher discrepancy than 7% between stated and measured dose [1]. The 1998 EQUAL program, auditing 102 centres, noted a rate of 3% of the photon beam outputs in reference conditions showing deviations outside the acceptance level of 5% [6] and the IAEA 1998–2001 postal results for 526 photon and 791 Co-60 beams revealed 16% of the beams having a discrepancy outside the 5% limit and even 1.3% (17 beams) with a discrepancy of more than 20%, with, in general, worse results for the Co-60 beams than for the photon beams [7]. Other old reports stemming from over 10 to 23 years ago also reported significant discrepancies [8–11]. Dutreix and co-workers found 37 out of 125 beams outside the 3% optimal level and observed that 16 out of 22 beams with deviations between 3 to 6% and all 15 beams with a deviation of more than 6% were from centres not participating in BOAs within the previous 5 years (8). Izewska and Andreo observed that Only 65% of those hospitals who received TLDs for the first time had results within the acceptance limits, while more than 80% of the users that had benefitted from a previous TLD audit were successful [9].

In the current results, covering reports coming throughout a much wider part of the world, only 0.2% of the photon beams were found to be higher than the acceptance level.

Another feasibility study by the European-Commission Network verifying mailed dosimetry for electron beams was initiated in the late nineties. In total 300 (91.5%) out of 328 beams were found to be within the tolerance level of 5% [12]. Currently only 0.7% of all the electron beams were found to be out of this range.

Overall good results could also be seen for the measurements in non-standard conditions. The percentage number of all photon beams – standard and non-standard conditions - within the non-optimal level of acceptance from the 1998 EQUAL program are 14% and those outside of acceptance level are 4%, while the presented results have 6.5 and 0.1% of the beams in these categories of acceptance [6].

The difference in our results to those found in literature might be caused by the presence of a bias, as our dataset only contains BOA-reports from centres participating in EORTC lead radiotherapy trials. Additionally, centres might have provided only a selection of their BOA data to the EORTC.

More recent publications on RTQA results in trials suggest that the main sources of protocol deviations are not due to beam output deviations under reference conditions. Deviations are more likely to be related to ambiguousness in the trial protocol, delineation variations and dose calculation and optimization problems in trial specific treatment plans [13]. Other trial RTQA procedures, which are more and more harmonized globally, like benchmark exercises, individual case reviews and complex dosimetry checks are able to detect such protocol deviations and nowadays mitigate the need for BOAs in the context of trial RTQA [4, 14].

It can be defended that omitting the request of a BOA would make trial RTQA easier, faster and less costly without loss of RT quality within trials.

## Conclusion

Our analysis shows that only 0.2 and 0.7% of all photon and electron BOA results, respectively, were found discrepant in respect to a 5% cutoff level, which is a substantial improvement compared to previous BOA results.
